# Psychology of Shakspeare

**Published:** 1861-11

**Authors:** 


PSYCHOLOGY OF SHAKSPEARE.—Little did the great English dramatist imagine, even supposing him to have indulged in the most sanguine expectations of posthumous fame, that his plays would be regarded not only as the noblest creations of genius and the finest and most truthful delineations of human character, but that they should come to be studied as an encyclopedia of philosophy, wit, botany, mental pathology, and even medicine. Mrs. Jamieson has given us the "Women of Shakspeare," another writer The Flowers spoken of by Shakspeare, and we have now to notice a volume whose title heads this article. It is written by Doctor Bucknill, one of the authors of "A Manual of Psychological Medicine, or of the History, Causes, etc., and Treatment of Insanity."

Although we do not sit in our critical chair at this time, but merely act the part of a sketcher, we cannot refrain from expressing our regret at the title of psychology applied to a study of the morbid states of mind portrayed by Shakspeare in many of his dramas. Dr. Bucknill defends himself by telling us: "The derivation and original use of a term, however, not unfrequently differ from its acquired and permanent use, and the term psychology has, of late years, been used to denote all that relates to the department of science which takes cognizance of irregularities and aberrations and diseases of the mind." We can only account for this peculiar notion of the restricted application of the study of psychology by the author, to the probable fact of his own line of reading leading him so much in one channel as to make him overlook the claims of a wider philosophy for that which he would thus appropriate exclusively to a particular purpose. Psychology, as teaching the laws and relations of the changes and phenomena which take place in the mind during the intellectual operations, is not more the study of morbid or unsound states of mind, than physiology is of morbid states of the functions of the body. Feuchtersleben, in his erudite work on medical psychology, tells us that the foundations of this science are philosophy and physiology, which treat, the former of spirit, the latter of the organism, while the subject of medical psychology is the relation of each to the other. He believes that animal psychology (zoo-psychology) may throw much light on human psychology, especially with regard to instinctive impulses. Mental education, according to the same writer, makes a part of medical psychology. In the proper and generally received acceptation of the word, we can readily speak of a psychical mode of cure, and of psychical remedies. Were we to admit Dr. Bucknill's gloss on the word, these phrases would imply morbid or pathological mode of cure and of remedies. In the treatment of a lunatic, we have not to deal with a patient in a psychological state, but with one on whose deranged mind we act by the judicious introduction of psychical remedies, viz.: of the affections, sentiments, and reasoning faculties, which have been appropriately called the dietetics of the mind.

But, in obedience to our first intention, we pass from the critical to the admiring mood, and follow gratefully the lead of Dr. Bucknill in his excellent analyses of the mental characters of several of the creations of Shakspeare. This wonderful genius himself, the pride and glory of his country, is called, in words which cannot be considered too strong, "the greatest poet of all ages and pre-eminently the most truthful analyst of human action. Shakspeare not only possesses more psychological insight than all other poets, but than all other writers, the sacred writings alone excepted. He has been aptly called 'a nature humanized.' He has, above all men, the faculty of unraveling human action. Compared with his profound knowledge of the surface and depths of the human soul, the information of other great minds, even of such wondrously vigorous intelligences as those of Plato and Bacon, were obscure and fragmentary." The number of characters to which Shakspeare has attributed disordered states of mind, and the extent to which he has written on the subject, show that it was a favorite study of his—"on no other has he written with such mighty power." His opportunities for observation in this line were great. When he wrote there was, with the exception of the poor and small establishment of Bethlehem Hospital in London, no asylum for the insane in the kingdom. The afflicted in this way were not secluded from the world as they are now. "If their symptoms were prominent and dangerous, they were, indeed, thrust out of sight very harshly and effectually; but if their liberty was in any degree tolerable, it was tolerated, and they were permitted to live in the family circle, or to wander the country. Thus every one must have been brought into immediate contact with every variety of mental derangement; and every one who sought the knowledge of their peculiarities would find it at every turn." It was reserved for the alembic of the great mind of Shakspeare to convert these opportunities into psychological science.

Dr. Bucknill, in these psychological essays, opens to us new views of the wonderful and various powers of Shakspeare, and has gone beyond any previous commentator in his searching analysis of the strong and overpowering passions exhibited in the pages of the great poet. Although we mark as quotations many passages which follow, we may as well tell our readers that the intervening remarks are also, with slight exceptions, given in his own words. His first selection is "Macbeth, the most awful creation of the poetic mind, and a study every way worthy of those to whom the storms of passion present the frequent cause of mental disease." This drama, "in the mental development of the bloody-handed hero and of his terrible mate, affords a study scarcely less instructive than the wild passionate madness of Lear, or the metaphysical motive-weighing melancholy of the Prince of Denmark."

"The great interest of this drama is most skillfully made to depend upon the conflicting emotions of sympathy with a man struggling under fearful temptation; horror excited by treachery and foul murder; awful amazement at the visible grasp of the Spirit of Evil upon the human soul; and of satisfied justice at the hell of remorse into which he is plunged."

"Macbeth is no villain in grain, like Richard the Third or Iago, rolling in the devil's work because he likes it; but a once noble human nature, struggling but yielding in a net of temptation, whose meshes are wound round him by the visible hand of the Spirit of Evil. Slave as he is to that soldier's passion, the love of fame and power, he is not without amiable qualities. He was once loved even by his arch-enemy Macduff, to whom Malcolm says,

'This tyrant whose sole name blisters
our tongues,
Was once thought honest; you have
lov'd him well.'

"Let it not be thought that we attempt to palliate the guilt of Macbeth. In a moral point of view, this is impossible. If his solicitings to crime are supernatural, combined with fate and metaphysic aid, he is not blinded by them. With conscience fully awake, with eyes open to the foul nature of his double treachery, although resisting, he yields to temptation. He even feels that he is not called upon to act to fulfill the decrees of destiny:—

'If Chance will have me king, why Chance may crown me
Without my stir.'

"Had he with more determination resisted the temptations of the woman, he might have falsified the prophecies of the fiend and put aside from his lips the poisoned chalice of remorse, maintained from rancors the vessel of his peace, and, above all, have rescued the eternal jewel of his soul."

Dr. Bucknill does not, as a "mental physiologist," withhold his belief in influences of the supernatural event, which is not only the starting-point of the action, but the remote cause of the mental phenomena. The professed moralist is slow to accept the teaching of the drama; but where shall we find a more impressive lesson of the manner in which the infraction of the moral law works out its own punishment than in the delineation and agonies of the torture of Macbeth? The fiercer temptation in the solicitings of the evil one, and the agency of Macbeth's terrible human tempter and colleague, his wife, are prominent impellers in his course to destruction. The extreme excitability of his imagination must not be overlooked, as when the supernatural soliciting of the weird sisters suggests to him an *image*, not a thought, so horrible that its contemplation

"Does unfix my hair
And make my sealed heart knock at my ribs,
Against the use of nature."

"This early indication of Macbeth's tendency to hallucination is most important in the psychological development of his character."

The progressive steps of the temptation, held out by Macbeth's wife, to commit the bloody deed, are well indicated in the essay before us. The woman herself has no faltering. Ambition and the desire "of sovereign sway and masterdom" are, to her undaunted metal, the all-sufficient motives of the terrible deed which she plotted and instigated, and would have perpetrated had not a touch of filial piety withheld her hand. The waverings of Macbeth in his first soliloquy indicate a sensitive appreciation of a right motive, as does the expression of sincere pity for the gracious Duncan. When Lady Macbeth joins him, he expresses his virtuous resolve, and for the first time adds "prudential reasonings." Then, continues our appreciating critic, mark the temptation to which the terrible woman subjects him—the taunts of cowardice and weakness, taunts to which a soldier gifted with personal bravery would be keenly alive, especially coming from the lips of a beautiful woman whom he loved. She further urges the temptation by comparing his vacillating desire with her own fell purpose, in that terrible passage beginning with "I have given suck," etc. Fearing that his better nature would relent, she had sworn him to the treacherous and bloody deed. She concludes by showing clearly the opportunity.

"He reels under the fierce battery of temptation, and when she has poured her spirit into his ear and chastened his compunctions with the valor of her tongue, he falls."

"The deed is done; and the terrible punishment of guilt dogs the murderer's heels even from the chamber of death. Guilt hath instantly changed the brave man into the coward. The sting of remorse extorts from him the direst expression of regret, which is in strong contrast with the woman's firmer nerve rebuking him."

The passage "Methought I heard a voice," is, in Dr. Bucknill's opinion, scarcely to be accepted as another instance of hallucination: it is rather one of merely excited imagination without sensual representation. "How exquisite is this description of sleep! How correct psychologically is the threat that remorse will murder sleep! How true the prediction to the course of the drama, in which we find that, hereafter, the murderer did 'lack the season of all natures, sleep!'" How awful is the retribution which the Nemesis of conscience works upon the guilty pair, and that before they have come to dread any earthly retribution! It is now the woman's turn to suffer. Listen to the deep sound of melancholy surging from her heart—

> "Naught's had, all's spent
> Where our desire is got without content;
> 'Tis safer to be that which we destroy
> Than by destruction dwell on doubtful joy."

The banquet scene, following the murder of Banquo, is unrivaled in dramatic force and psychological truth. The hallucination at the banquet, in which Macbeth fancies he sees the ghost of the murdered Banquo, is admirably wrought out. Unlike the air-drawn dagger, which he recognized as an hallucination, he believes this appearance to have been most real. He is at this juncture in a state of mind bordering on disease, if he has not actually passed the limit. Truly the caution given by his wife was likely to become a prophecy—

> "These deeds must not be thought on
> After these ways; so it will make us mad."

The fear was in reality, after awhile, fulfilled in the instance of the woman. Macbeth, however, saved himself from actual insanity by rushing from the maddening horrors of meditation into a course of decisive, resolute action. Henceforth he gave himself no time to reflect; he made the firstlings of his heart the firstlings of his hand; he became a fearful tyrant to his country; but he escaped madness. The rapid deterioration of Macbeth's moral nature deserves notice. In the assassination of the kindred of Macduff we find him committing a wholesale and motiveless deed of blood, thus passing from infirmity of purpose, with conscientious rebukes for the murder of the king, to the massacre just mentioned, without hesitation or remorse. Subsequently to this foul deed the tyrant supported his power with many acts of sudden and bloody violence.

"The character of Lady Macbeth is less interesting to the psychological student than that of her husband. It is far less complex; drawn with a classic simplicity of outline, it presents us with none of those balancing and contending emotions which make the character of Macbeth a wide and varied field of study." "The terrible remorseless impersonation of passional ambition delineated in the character of Lady Macbeth, is not gradually developed, but is placed at once in all its fierce power before us in that awful invocation to the spirits of evil."

The turning-point of her madness was the state of inactivity into which she fell, when her husband broke away from her support into that bloody, bold, and resolute career which followed the murder of Banquo. In Macbeth's more demonstrative and flexible nature passion was explosive; in her's it was consuming. In him the inward forces found a volcanic vent; in her their pent-up force shook in earthquake the deep foundations of the soul.

"Lady Macbeth's end is psychologically even more instructive than that of her husband. The manner in which even-handed justice deals with her, 'his fiend-like wife,' is an exquisite master-piece of dramatic skill. The undaunted metal which would have compelled her to resist to the last, if brought face to face with any resistible adversaries, gradually gives way to the feeling of remorse and deep melancholy, when left to feed upon itself. The moral object of the drama required that the fierce gnawing of remorse at the heart of the lady should be made manifest; and as her firm, self-contained nature imposes upon her a reticence in her waking moments in strong contrast to the soliloquizing loquacity of her demonstrative husband, the great dramatist has skillfully availed himself of the sleep-talking state in which she uncovers the corroding ulcers of her conscience."

The diagnosis arrived at by the "Doctor," who is introduced in the scene, appears to have been that she was scarcely insane, but so sadly troubled in conscience as to be prone to quit the anguish of this life by means of suicide.

> "Unnatural deeds
> Do breed unnatural troubles; infected minds
> To their deaf pillows will discharge their secrets.
> More needs she the divine than the physician.
> God, God, forgive us all! Look after her;
> Remove from her the means of all annoyance,
> And still keep eyes upon her."

A passage at the very end of the drama indicates, though it does not assert, that the fear of the Doctor was realized.

> "His fiend-like queen
> Who, as 'tis thought, by self and violent hands
> Took off her life."

The contempt of physic expressed in the oft-repeated language of Macbeth—

> "Throw physic to the dogs, I'll none of it,"

gives rise to some judicious remarks by Dr. Bucknill, in which he refers to the ignorance of the leech and the mediciner of former times, who had not learned to combine the moral influences which are the true means of ministering to a mind diseased, after the manner of Lady Macbeth, with those sleep-producing oblivious antidotes, which at present form the remedies of melancholia. Such would be the course pursued by a physician of the present day—certainly by any man who would deserve to be called a psychological physician.

Dr. Bucknill, after putting the question, "What was Lady Macbeth's form and temperament?" criticises Maclise's representation of her in the painting of the banquet scene, as a woman of large and coarse development, a Scandinavian amazon. According to the author's conception, Lady Macbeth was a lady, beautiful and delicate, whose one vivid passion proves that her organization was instinct with nerve force, unoppressed by weight of flesh. Probably she was small; for it is the smaller sort of women whose emotional fire is the most fierce, and she, herself, bears unconscious testimony to the fact that her hand was little. The drama contains many indications, that to outward appearance she was gentle and feminine.

Dr. Bucknill, in the same spirit of appreciative analysis, reviews the character of *Hamlet* as elaborated by Shakspeare. He says, most truly, that all critical study of Hamlet must be psychological, and although criticism upon it may seem to be exhausted, yet "some leavings of treasure will be discoverable to those who seek it in an earnest and reverent spirit." It is known that Shakspeare devoted more time to this piece than to any other of his works, and that in its construction he altered and realtered it much.

"It was the labored and elaborate result of years of toil, of metaphysical introspection, and observation. It was the darling child of its great author, and ran some risk of being a little spoiled."

The first scene, where the Ghost appears to the sentinels on watch, is constructed with exquisite dramatic verisimilitude. It takes possession of the mind and the imagination at the commencement. Hamlet is from the first represented in that mood of melancholy which vents itself in bitter sarcasm. In his pure and sensitive mind the conduct of his mother has produced shame and keen distress. In his passionate outburst of grief, given by the poet, we see the conflict between religious belief and suicidal desire, in which the former prevails. The early passage beginning "Thrift! thrift! Horatio," "seems to give the key-note of Hamlet's temper, namely, soul-crushing grief in close alliance with an inimical, often a broad humor, which can mock at despair." The courage of the Prince is of the noblest temper. After the ghost has told the terrible tale and has disappeared, Hamlet becomes wild with the feeling of revenge; but in a moment he passes from the terrible to the trivial—a state and a transition of mighty emotion into lowliness of action, which Dr. Bucknill asserts is "one of the finest psychological touches anywhere to be found in the poet."

"How it is that the resolution of Hamlet to put on the guise of madness follows so quick upon the appearance of the ghost to him, (indeed while the spirit is yet present, though unseen, for the intention is expressed before the final unearthly adjuration to swear,) we are unable to explain."

The purport of his feigned madness is not very obvious. It was, to use the language of Schlegel, acted with inimitable superiority. There are those who believe that the Prince of Denmark was actually crazed, and who cite as one strong proof of the fact his harsh and cruel treatment of Ophelia, whom he really loved. Dr. Bucknill sums up the mental condition of Hamlet in the following words:—

"His conduct to Ophelia is a mixture of feigned madness, of the selfishness of passion blasted by the cursed blight of fate, of harshness which he assumes to protect himself from an affection which he feels hostile to the present purpose of his life, and of that degree of real unsoundness, his unfeigned 'weakness and melancholy' which is the subsoil of his mind."

In reference to his feigned insanity, the author remarks:—

"The characteristics of this feigned form are those of mania, not indeed violent, acute, and demonstrative, but mischievous, reckless, and wayward, and so mingled with flashes of native wit, and disguised by the ground color of real melancholy, showing through the transparency of the feigned state, that Hamlet's character becomes one of the most interesting and complicated subjects of psychological study anywhere to be met with."

But we find that we must forego the pleasure of being any longer the medium between our readers and the psychological critic and delineator of Hamlet, and draw to a close our repetitions of his text and glosses of his loving and subtle study of the great dramatist. Of the next and kindred essay on Ophelia, we cannot do more than introduce her, in the words of Dr. Bucknill, as a

"Child of nature in simplicity and innocence; without guile, without suspicion, and therefore without reserve, or that degree which often simulates a modesty more dainty than the modesty of innocence." "She says nothing of herself, and yet we seem to look into the very recesses of her clear soul; thus presenting one form of contrast to the being with whose fate her own was entwined, who, constantly soliloquizing and self-analyzing, nevertheless leaves upon us the impression that we know the vast amplitude of his thoughts and feelings but dimly and apart." "Sensitive and imaginative, and devoted, the poor girl was endowed with all the faculties of moral suffering. That she should suffer greatly, undeservedly, irremediably, was needful in order to make her the object of that intense pity which the character excites; and which was certainly wanted in the drama to perfect it as a tragedy. The character is not very prominent, but it so entirely seizes upon our sympathy and pity, that in this respect it leavens our regard for the whole play."

The portrait of Ophelia drawn by the author is in the very best style of art, and would alone commend the entire volume to the regards of the reader.

"King Lear" follows in this gallery of dramatic paintings.

"Oppressed by the power and magnitude of the passions, as depicted in this most sublime and awful of poetic creations, it is only after the senses have become accustomed to the war and turmoil that we throw off the stupor, and dare to look down upon the throes of Titan, and begin to recognize the distinctive features of the fierce commotions."

A new field of psychological investigation thus opened is surveyed and scanned by Dr. Bucknill in every part, and with a keen and discriminating eye to its rich and rare productions.

Timon of Athens is the next in the list of the characters for psychological study. To this succeed Constance, Jaques, Malvolio, Christopher Sly, Comedy of Errors. Such an enumeration cannot fail to pique a curiosity to which in degree we have ministered, in bringing out a part of the rich material furnished by the author in his essays on Macbeth and Hamlet. We have shown, however imperfect this sketch of ours may be, that, notwithstanding the many critical commentaries on Shakspeare by such men as Johnson, Stevens, Hazlitt, and Coleridge among the English, and Schlegel and Goethe among the German scholars, Dr. Bucknill, by his Psychology of Shakspeare, may claim a place, and that not an inferior one, in this illustrious company, for giving us new insight into the creations of the great dramatist and into human nature, of which there are so many striking varieties.

